# Disorders of the Reproductive Health of Cattle as a Response to Exposure to Toxic Metals

**DOI:** 10.3390/biology10090882

**Published:** 2021-09-08

**Authors:** Marcjanna Wrzecińska, Alicja Kowalczyk, Przemysław Cwynar, Ewa Czerniawska-Piątkowska

**Affiliations:** 1 Department of Ruminant Science, West Pomeranian University of Technology, ul. Klemensa Janickiego 29, 71-270 Szczecin, Poland; marcjanna.wrzecinska@zut.edu.pl (M.W.); ewa.czerniawska-piatkowska@zut.edu.pl (E.C.-P.); 2 Department of Environment Hygiene and Animal Welfare, Wrocław University of Environmental and Life Sciences, Chełmońskiego 38C, 51-630 Wrocław, Poland; przemyslaw.cwynar@upwr.edu.pl

**Keywords:** toxic metals, cattle, fertility, reproduction

## Abstract

**Simple Summary:**

Heavy metal pollution is common in the environment and can come from natural sources such as forest fires and volcanic eruptions, as well as from anthropogenic sources: mines, smelters, or refineries. These elements are toxic to living organisms and internal organs and can accumulate in living organisms. They can negatively affect both female and male fertility. Chronic exposure of cattle to toxic metals can cause embryotoxicity, disturbances in spermatogenesis, and oocyte development. It is important to monitor environmental pollution with toxic metals.

**Abstract:**

The aim of this review is to comprehensively present disorders of the reproductive system in cattle exposed to contact with toxic metals. Toxic metals are a common environmental pollutant and can come from mines, smelters, fossil fuel combustion, or volcanic eruptions. Metals have the ability to bioaccumulate in living organisms, thus contaminating the food chain and may pose a threat to humans. They accumulate mainly in the liver and kidneys, but also in muscles and fat tissue. Toxic metals such as lead (Pb), arsenic (As), mercury (Hg), and cadmium (Cd) have a negative impact on the fertility of animals; they can lead to abortions, premature calving, or oocyte dysfunction. Moreover, in the male reproductive system, they disrupt spermatogenesis, and cause apoptosis of sperm and oxidative damage. The main source of exposure of livestock to toxic metals is through the consumption of feed or contaminated water. It is important to monitor the level of heavy metals in animal products to prevent human poisoning. Toxic metal biomonitoring can be performed by testing urine, blood, milk, plasma, or hair. Chromium (Cr), arsenic (As), and cadmium (Cd) are excreted in the urine, while lead can be detected by examining the blood of animals, while in milk, arsenic (As), cadmium (Cd), nickel (Ni), and lead (Pb) can be detected. Moreover, toxic metals do not biodegrade in the environment. To purify soil and waters, remediation methods, e.g., biological or chemical, should be used.

## 1. Introduction

The presence of toxic metals in water and animal feed is dangerous for animals and humans due to their bioaccumulation. They are a threat to human health as they can cause the inhibition of kidney function disorders and diseases of the cardiovascular and nervous systems [1]. The aim of this review is to comprehensively present disorders of the reproductive system in cattle exposed to contact with toxic metals.

Agricultural production (mainly the production of ruminant meat and milk), after the energy industry, is responsible for the largest emissions of greenhouse gases, having an adverse impact on the environment [2]. According to Bonnet et al. (2018) [2], the average global meat consumption increased by 60% between 1990 and 2009, and this increase is continuing. In Europe, as one of the largest meat consumers, there is also an increase in meat consumption. The highest consumption of meat is recorded in Germany, France, Great Britain, Italy, and Spain [2]. In Australia, the annual meat consumption is 116 kg per capita, the largest meat consumption of any country in the world. The United States is next in terms of meat consumption (over 110 kg per year), then Europe (about 80 kg of meat) [3].

FAO has released a report that world meat production has dropped to 325 Mt in 2019. The main reason of this was African Swine Fever (ASF) in China and Europe [4]. FAO forecasts an increase in world meat production by nearly 40 million tons by 2029, consequently reaching 366 million tons. According to these estimates, the demand for pork and beef will decline in the European Union. In America, including the US, beef production will increase until 2029, while in Asia there will be an increase in demand for pork, related to the recovery after African Swine Fever (ASF). Moreover, FAO forecasts an increase in poultry production due to the short production period and the efficient possibility of improving the genetics of animals. This increase will occur mainly in China, the USA, and in some European countries, such as Poland and Romania. Mutton production will increase significantly in Africa and China [4].

A serious food safety problem is the presence of toxic metals in meat, a small amount of which is a hazard to humans [5]. Elements classified as toxic metals are lead (Pb), mercury (Hg), cadmium (Cd), arsenic (As), zinc (Zn), cobalt (Co), chromium (Cr), manganese (Mn), iron (Fe), barium (B), lithium (Li), zirconium (Zr), selenium (Se), molybdenum (Mo), aluminum (Al), and copper (Cu) [6,7]. Meat and meat products are contaminated with metals mainly from the environment, as well as from industrial waste. Contamination with lead and mercury occurs most often in the case of environmental pollution, usually from mines, or waste gases, as well as pesticides and herbicides used on pastures [5]. According to Das et al. (2021) [8], livestock may be a threat to the toxic effects of arsenic through the consumption of animal products and meat. It was found that, in cows exposed to arsenic, this metal is excreted into their milk. Arsenic has also been detected in contaminated areas in chicken eggs, and chicken, goat, duck, and cow meat [8]. The latest EFSA report [9], identified that the human exposure to arsenic occurs via dermal and inhalation exposure, but also via food and drinking water [9]. It is also found that the reference point between 0.3 and 8 μg/kg bw per day is a benchmark dose and lower confidence limit (BMDL01) for a 1% increased risk of lung, skin and bladder cancers, as well as skin lesions [10]. There are no generally accepted safe levels of arsenic presence in food, including products of animal origin and there is a lack of unified international standards for this toxic metal. Nevertheless, the WHO sets an acceptable arsenic intake of 3.0 μg/kg body weight [8]. Water intended for human consumption in the EU has a parametric value of 10 μg/L arsenic, different from what is related in EU legislation [9,11]. As it was noted by the FAO/WHO report (2018), Codex Alimentarius established various total levels of arsenic forms in different food products (10 μg/L for natural mineral water; 100 μg/kg for edible fats, oils, fat spreads and blended spreads; 500 μg/kg for food grade salt; or 200 μg/kg for polished rice and 350 μg/kg for husked rice) [9,12]. Das et al. (2021) [8] found that the arsenic content of animal products was higher in contaminated areas than in unpolluted areas. It was found that As is detected in cow’s milk and in poultry liver, which is characterized by a greater arsenic deposition than meat [8].

World milk production is projected to increase by 1.6% per year by 2029 (to 997 Mt) due to the optimization of milk production processes, and improvements to animal health and productivity [13]. According to FAO and OECD (2020) [13], India and Pakistan will make a significant contribution to this growth. In Europe, milk consumption in 2019 was 64.9 kg per capita, and in the USA it was 63.96 kg [14]. Milk and dairy products are valuable sources of macro- and micro-nutrients, as well as the basic product for the nutrition of children. Milk can be contaminated with toxic metals, such as lead, cadmium, arsenic, and mercury [15], but also chromium and nickel [16,17]. The toxic metals in milk pose a danger to humans and the contamination of the milk is an important issue. These toxic metals enter into the milk through contaminated feed and water, most often in industrialized areas, near steel mills and mines [18]. The source of contamination can be containers of milk, feed, or the animal environment [15].

A study in Jersey cows confirmed the bioaccumulation of toxic metals in milk. Somasundaram et al. (2005) reported that the Pb, Cd, Ni and Cr ranged in milk from minimal levels up to 0.046, 0.056, 35.48 and 22.5 mg/kg, respectively. The upward trend of Pb was also observed in the first 7–10 days of the experiment, when its presence was noted within the permissible limit of 0.1 mg/L. The research showed a doubled Cd level, which was at a constant value after 10 days of the study [17]. Nevertheless, it was found that the Cr level in Jersey cow milk was characterized as the highest and the longest concentration in the group of analyzed toxic metals (22.5 mg/kg in 22 day). In the research conducted by Das et al. (2021) [8], the average arsenic content in cow’s milk from the area exposed to contamination with toxic metals was found to be over 6 µg/L (double the value of the control group). Moreover, milk contains inorganic arsenic, while methylated arsenic does not pass through the udder epithelium of cows. Furthermore, Das et al. (2021) [8] indicated that the accumulation of arsenic in milk mainly occurs in casein (83%), then in fat (10%), in whey protein (4%), and in skimmed milk (3%). Casein, due to its structure of numerous phosphate groups and phosphoserine units, binds arsenic in these units [8].

**Figure 1:**
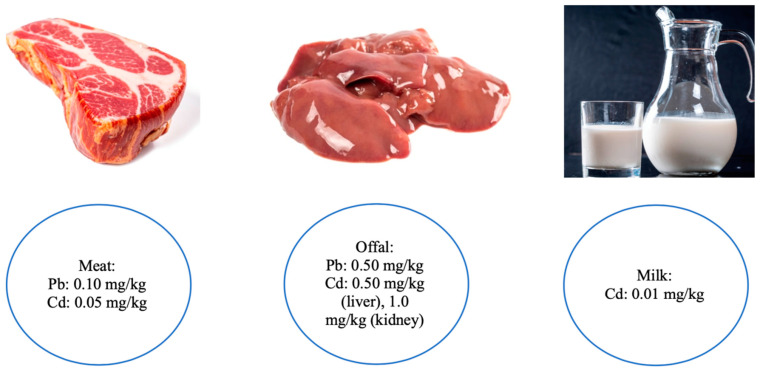
Permissible content of lead and cadmium in animal products in UE. Source: European Union. 2006. Commission regulation (EC) no. 1881/ 2006 setting maximum levels for certain contaminants in foodstuffs; https://www.flickr.com/photos/30478819@N08/51118318084 (Creative Commons 2.0, 1 April 2021); https://www.flickr.com/photos/30478819@N08/50530382173 (Creative Commons 2.0, 1 October 2020); https://www.flickr.com/photos/30478819@N08/31463958607/in/photostream/ (Creative Commons 2.0, 1 December 2018).

The figure below (Figure 1) shows the maximum permitted concentrations of cadmium and lead in animal products in the UE.

Urbanization results in the deterioration of environmental pollution, as well as inadequate waste management. The use of pesticides and artificial fertilizers leads to environmental pollution with toxic metals. They pollute the soil, plants, and, subsequently, meat, which is a threat to humans [5].

## 2. Toxic Metals and Sources

**Figure 2:**
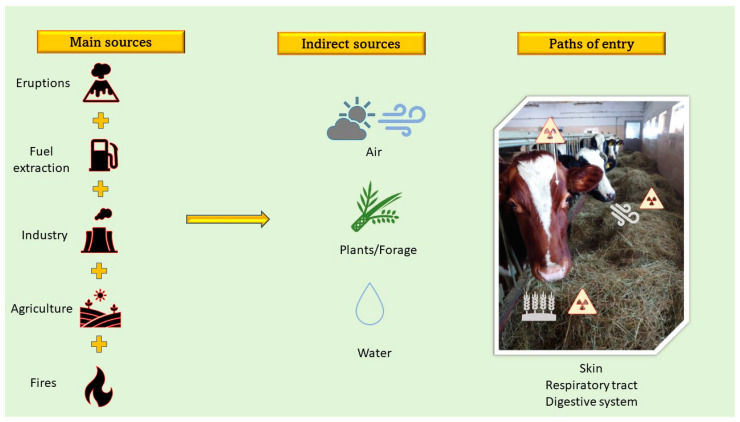
The main sources of toxic metals contamination and the paths of their entry into the body.

Toxic metals show an embryotoxic, a hepatoxic, a nephrotoxic, as well as a carcinogenic and mutagenic effect on living creatures [6]. They are non-biodegradable and widespread in the environment [6,19]. However, the most toxic to animals and humans are lead, cadmium, mercury, and arsenic [7]. These metals enter the environment from two sources [7]. On the one hand, they come from an anthropogenic origins, mainly in the presence of coal mines, smelters, and refineries, but are also found in pesticides, and from natural source, for example, during volcanic eruptions [7]. It was already proven [20,21] that the non-ferrous metal industry causes the long-term degradation of the natural environment as well as the ground waters, agricultural ecosystems, and food safety, causing serious risks for plants, animals and humans. Moreover, as it was pointed out by Ghazaryan et al. (2016) [21], the contamination of soils by heavy metals is causing huge concern due to the potential effects on human health and the possible long-term sustainability of food production in contaminated areas, since it is well known that contaminants circulating in the environment can pass through food chains. Tailings ponds and the high toxicity related to this industry is a serious problem, not only because of their primary constituents but also as a result of the chemicals used during ore processes [20,22]. Nevertheless, industrialization has significantly contributed to the emission of pollutants into the natural environment, including water and soil. Toxic metals contained in soil, which are toxic metals, penetrate into growing plants, where they can be accumulated [7]. During grazing or feeding, animals with fodder contaminated with toxic metals or toxic compounds, enter into the animals’ organisms [7,23]. Toxic metals enter into the animal body through the respiratory and digestive systems or through dermal contact [7,23] (Figure 2). It should be noted that the high concentration of numerous trace elements (incl. Fe, Mn, Zn, Pb, Cd, Cr, Hg or Co) may be a reason for the functional and physiological deficits of animal and human organisms such as Minamata (Hg), Itai-Itai sterility (Cd), Kesan (Se deficiency) or Wilson’s disease (Cu toxicity) (17).

In animals, toxic metals bioaccumulate in tissues, mainly in the liver and kidneys [23]. The concentrations of cadmium and lead in the liver and kidneys of calves are usually higher in industrial areas than in rural areas, which may affect the metabolism of other elements and the health of the calves through the effects on the endocrine system [23].

## 3. Fertility

Toxic metals, such as lead, arsenic, mercury, and cadmium, can affect animal fertility, for example, gametogenesis in cattle and in other ruminants [6,19]. Furthermore, there are also some elements that influence the biological functions of animals: chromium, copper, iron, selenium, nickel, zinc, manganese, molybdenum, and cobalt, among others (Figure 2) [6]. The toxic effect of the compound depends on the ingested dose and the time of exposure [24,25]. Toxic metal pollution has an impact on human and animal health [24].

In the female reproductive system, toxic metals accumulate in the follicular fluid and damage the ovarian granulosa cells, leading to impaired hormone synthesis, as well as reducing the quality of oocytes [24]. Moreover, toxic metals can lead to pregnancy loss or premature calving. They can also cross the placenta to reach the fetus and cause developmental problems [7,8].

Toxic metals in the male reproductive system may contribute to male infertility, because these compounds disturb spermatogenesis, cause the apoptosis of sperm, and oxidative damage [6,7]. These elements accumulate mainly in the testes, epididymis, vas deferens, seminal vesicles, and in semen [6]. Bulls exposed to toxic metals showed a decrease in sperm count and poorer semen quality [6]. Moreover, the exposure of animals to toxic metals causes necrosis, hemorrhage, or germ cell losses in calves [6].

### 3.1. Disorders of Gametogenesis

Numerous studies have shown that exposure to toxic metals is associated with disturbances of gametogenesis [6,20,26,27].

In cattle, lead contributes to induced infertility. The accumulation of lead in the alveolar fluid of cows significantly reduces folliculogenesis [28]. In studies on mice, 10 mg/kg of lead was administered for 15 weeks; impaired folliculogenesis and an increase in atretic primary follicles were observed [29]. In males, lead reduced spermatogenesis through Leydig cell atrophy, thereby reducing the density, quantity, and quality of semen and some semen components, such as fructose [7,27]. Then, fructose reduced the content of succinate dehydrogenase and alkaline phosphatase, which affected the development of abnormal sperm, including azoospermia, asthenozoospermia and morphological abnormalities [7].

In bulls, arsenic impairs spermatogenesis and the secretory functions of Sertoli cells. It can also damage the tissues of the testicles [7]. Mercury also reduces fertility by limiting spermatogenesis through the inhibition of tissue function in the testes and spermatogenic cells [7,25,30]. In addition, arsenic also causes damage to the testicular tissues. Arsenic causes hyperplasia of testicular interstitial cells and disorders of spermatogenesis by reducing the level of gonadotropins and limiting testosterone synthesis [7]. Similar effects are shown via exposure to cadmium. It causes interstitial damage to the testes and epididymides, which contributes to impaired spermatogenesis [31].

### 3.2. Gamete Dysfunction

Toxic metals have a negative effect on reproductive cells; they reduce the quality of oocytes and sperm [6,24]. These elements lead to the occurrence of male infertility through the ability to deregulate long noncoding RNAs in sperm and testes [6]. In vitro studies in calves, rabbits, and rodents have shown that the acute exposure to heavy metals results in testicular necrosis and swelling, hemorrhage, and infertility due to the disruption of the blood–testicular barrier [6]. Studies on bulls exposed to toxic metals, including cadmium, showed a decrease in sperm count and a deterioration in sperm quality. The spermatozoa were characterized by a reduced viability and damage to the cell membranes [6]. Moreover, the presence of cadmium in the testes and the plasma of semen results in the increased peroxidation of membrane lipids, contributing to the reduction of motility [6]. It has been shown that in the male reproductive system cadmium damages the cell membrane and the DNA of sperm, limits their motility, and impairs acrosomal reactions [6]. In rats, cadmium poisoning alkalinizes epididymal fluid, which affects sperm motility [31]. According to Zhao et al. (2017) [31], cadmium impairs fertilization by reducing sperm motility, which limits the penetration of the oocyte by the sperm.

Cadmium affects the maturation of bovine oocyte and embryo development because it has a cytotoxic effect [19]. Depending on the concentration, this element has a different effect on the fertility of cows. Higher concentrations significantly reduce the viability and maturation of oocytes, leading to their death. According to the in vitro research on bovine oocytes by Akar et al. (2018) [19], a concentration of 2.0 and 20.0 μM CdCl2 after 24 h of incubation showed a negative effect on oocyte maturation, development, and morphological abnormities of oocytes, leading to their death. Moreover, higher amounts of cadmium (2.0 and 20.0 μM CdCl2) absorbed by bovine oocytes and blastocysts cause damage of the oocyte nuclei and the morphological defects of the blastocyst [19].

Lead and cadmium contribute to an increase in the oxidative stress of ruminant semen [6]. Oxidative stress occurs when there is an imbalance between reactive oxygen species (ROS) and antioxidants [32]. The negative effect on semen quality has the effect of oxidative stress and reactive oxygen species (ROS). Unsaturated fatty acids in the sperm cell membrane are exposed to ROS, which generate lipid peroxidation (LPO), which negatively affects sperm and leads to structural disturbances in the sperm acrosome region, which reduces the quality of the sperm and causes problems with fertilization [33,34]. Moreover, during oxidative stress, it is possible to generate reactive oxygen species that reduce sperm viability [34]. According to Llamas Luceño et al. (2020) [33], DNA fragmentation leads to the demethylation of sperm DNA, which inhibits gene expression and may also lead to a change in chromatin configuration. Sperm cells are very susceptible to this type of stress due to their poor antioxidant protection [32]. The movement of sperm flagella is disturbed and the permeability of the cell membrane is disrupted, because polyunsaturated fatty acids contained in the cell membrane are prone to lipid peroxidation, causing a reduction in vitality and motility [32] and reducing the fertility of bulls [6]. Moreover, according to Aglan et al. (2020) [28], oxidative stress caused by exposure to Pb leads to apoptosis and the impaired proliferation of granulosa cells.

Another metal significantly limiting the function of gametes is lead, the poisoning from which reduces sperm motility [29]. Pb poisoning in cattle leads to disorders in prostatic function, including prostate hyperplasia and cancer, as well as a reduction in sperm motility [28]. Moreover, rats showed a decrease in testicular weight, reduced sperm motility and viability with a dose of lead acetate of 20 mg/kg, taken for 56 days [29]. In research in rats, it was found that after 30 days of ingestion of 100 µg of lead, the animals showed irregular oestrus cycles and ovarian cysts [29]. Moreover, it was possible to transfer lead to milk through lactation [23]. In turn, in studies conducted on mice, reproductive disorders were observed in animals after the chronic exposure to lead contamination [7]. In these studies, mice were administered 10 mg/kg of lead for 15 weeks, which impaired folliculogenesis and the growth of atretic primary follicles [29].

Damage to sperm cell membranes is also caused by mercury, the main mechanism of action of which is to induce oxidative stress in sperm, which contributes to the reduction of fertility by damaging the gametes [25,30]. Disorders of sperm cell membranes caused by oxidative stress are associated with reduced sperm motility and the reduced ability to fuse with the oocyte, as well as damage to the genetic material of the gamete [30]. In bulls, as a result of mercury poisoning, there are losses in the testicular tissue and a reduction in sperm quality through the production of morphologically changed sperm, as well as the possibility of male cryptorchidism [7,30].

In females, mercury leads to a reduction in oocyte maturation [30]. Moreover, it has been observed in hamsters that exposure to mercury disturbs oestrus cycles, inhibits follicle development and lowers progesterone levels [7].

### 3.3. Fetal Abnormalities/Stillbirths

The described toxic metals are characterized by the ability to overcome the placental barrier and pass to the fetus, which is often associated with stillbirths, miscarriages, and disturbances in the development of the embryo/fetus [6,7]. It has been proven that arsenic passes through the placenta to the fetus, then the fetus is exposed to this toxic compound, which poses a risk of stillbirth, fetal deformity and multiorgan damage in the developing fetus [8]. However, in cows, lead contributes to induced infertility, premature calving, as well as abortion in pregnant cows [28].

Moreover, in cows, it has been shown that Hg readily crosses the placenta and can affect the fetus by causing fetal malformations and even miscarriages [7]. Thus, mercury is toxic to the fetus [7]. Cadmium is also fetotoxic to the developing embryo [19]. Research in rats showed that the exposure to cadmium at concentrations of 3, 10 and 30 ppm in water for 28 days changes the absolute myometrial tension. This element accumulates in the myometrium, blocks calcium channels, and modifies the muscle response to oxytocin [29]. Similarly, in pregnant cattle, cadmium also has the ability to block calcium channels in the myometrium, inhibiting muscle contractions [24]. Furthermore, exposure to cadmium can lead to premature calving. It also impairs placental function and reduces progesterone levels, and calves whose mothers are exposed to Cd are characterized by a low weight [7]. Moreover, in studies on mice (200 ppm of Cd in water, for 30 and 60 days), changes in the thickness of the myometrium and an increase in apoptosis in the endometrium after 60 days are noted, which may affect the implantation of embryos in the uterus [29].

### 3.4. Disturbances in the Synthesis of Reproductive Hormones

Toxic metals reduce the synthesis of sex hormones, which are crucial for normal fertility indicators [7,24]. It has been proven in mice that arsenic influences the formation of cystic uterine hyperplasia, which results from disturbances in estrogen secretion [7]. This metal also reduces testosterone levels in males and increases the stress hormone [6]. Moreover, researches have shown that Hg inhibits steroidogenesis resulting in a reduction in testosterone levels [7]. Another metal disturbing the hypothalamic-pituitary-gonadal axis is lead, which reduces the synthesis of reproductive hormones and thus disturbs the oestrus [28]. In hamsters, it is observed that mercury exposure disrupts oestrus cycles, inhibits follicle development, and reduces progesterone levels. This is also fetotoxic [7]. In research in rats, it was found that after 30 days of ingestion of 100 µg of lead, the animals showed irregular oestrus cycles and ovarian cysts [29].

Table 1 shows other toxic metals along with the site of their accumulation and influence on reproductive functions.

## 4. Toxic Effects of Metals

Exposure to toxic metals such as As, Cd, Pb, Hg is associated with an increased risk of diabetes, atherosclerosis, cardiac arrest and hypertension, which are risk factors for cardiovascular disease (CVD) [43,44]. These metals increase the risk of CVD by inducing endocrine disruptions and creating reactive oxygen species in the myocytes of the heart [43,44,45]. Toxic metals are characterized by oxidizing abilities. Studies in mice have shown that toxic metals increase oxidative stress and inflammation, which contributes to the formation of atherosclerotic lesions. Metals are responsible for the increased secretion of interleukin 6 (IL-6) or the tumor necrosis factor (TNF-a) [46].

Metals, such as mercury, lead, and cadmium are considered neurotoxic and promote the production of amyloid-β (Aβ) and the phosphorylation of the tau (P-tau) protein, which can cause the formation of amyloid plaques in the brain, a major pathology of Alzheimer’s disease (AD) [47]. Pb and Cd are responsible for the reduction of acetylcholine content in the brain, which is a key neurotransmitter directly related to the pathogenesis of AD. In turn, it has been proven that Hg can accumulate in the brain and cause oxidative stress and hence cell apoptosis, and is also involved in the pathogenesis of AD [47]. Meta-analyzes by Xu et al., (2018) [47] showed a significant increase in blood Hg and Cd levels in patients diagnosed with Alzheimer’s disease.

### 4.1. Cadmium

Cadmium is situated in the 12th group and 5th period of the periodic table. Cd is one of the most common environmental pollutants among toxic metals, and is carcinogenic [19,36]. The main sources of cadmium in the environment are Ni-Cd batteries, mining and smelting, and forest fires [36]. Cadmium bioaccumulates mainly in the lungs, kidneys, liver, bones, testes, epididymis, follicular fluid, and it enters the body through the consumption of feed and water [6,7,19,35,37]. A typical blood concentration of cadmium in livestock animals should not exceed 1 ppm [48]. This pollution has a negative impact on human and animal health. It induces oxidative damage to the amino acids, thereby creating unusual chemical bonds in these structures, which are called Advanced Protein Oxidation Products (APOPs). APOPs change the function and structure of proteins [49].

In vitro studies have shown that chronic cadmium exposure inhibits the mineralization of vertebral bodies, which results in a higher susceptibility to deformation and fractures [50]. Cadmium reduces the expression of markers of osteoblast differentiation, such as osteocalcin and Runx2, the latter of which is a type I collagen, forming the protein matrix of the extracellular bone, and reducing the action of enzymes involved in bone mineralization, such as alkaline phosphatase (ALP). Cd has also been shown to reduce bone volume and induce osteoblast apoptosis through DNA fragmentation and an increase in reactive oxygen species [50].

In vitro and in vivo studies have shown that cadmium generates reactive oxygen species by disrupting the activity of antioxidant enzymes such as catalase (CAT) and superoxide dismutase (SOD). According to Genchi et al. (2020) [45], Cd modulates the cell signaling pathway causing cell apoptosis. As shown in in vivo and in vitro studies, this metal induces epigenetic changes in mammalian cells, which contributes to the development of neoplasms [45]. In addition, there is an association between cadmium exposure and the occurrence of coronary heart disease, stroke, arterial disease, inflammatory reactions, or changes in the lipid profile [45,46].

### 4.2. Lead

Lead is the 6th element of the 14th column in the periodic table. This metal is released from mining gasoline from petrol and volcanic eruptions [36]. Pb enters into the animal body through the consumption of contaminated feed and water and accumulates mainly in the liver, kidneys, brain, and bones [7,36]. The physiological level of Pb in livestock animals’ blood is 0.24 ppm, but it has been noted that the concentration of 0.35 ppm is a diagnostic symptom of lead poisoning in cattle [48].

As has been proven, lead has the ability to mimic other metals, such as calcium and iron, therefore it binds and interacts with many enzymes, disrupting their activity [46]. During in vitro studies, lead has been shown to reduce the content of minerals in bones and affect the change in their properties, resulting in the formation of low-quality bones. The inhibition of osteoblast activity and the induction of their apoptosis caused by exposure to lead were also found [50]. Moreover, according to Kumar et al. (2020) [51], lead inhibits the activity of enzymes and cell systems, especially during cell development and hematopoiesis.

### 4.3. Arsenic

Arsenic is a metalloid placed in the 4th place of the 15th column in the periodic table. This element is released to the environment from many sources, for example, mining, smelting, combustion of fossil fuels, and volcanic eruptions [36]. It bioaccumulates mainly in the liver, kidney, heart, lungs, blood, and keratin tissues in hair [6,8,36]. It has the ability to transform to form arsenate (V) and arsenite (III), of which arsenite is up to 60 times more toxic [36,52] This is a carcinogenic and neurotoxic element [36].

In vitro studies have shown that arsenic has a negative effect on the bone formation process by reducing osteoblastic activity, as well as inhibiting the proliferation and induction of bone marrow mesenchymal cells (BMSC), osteoblasts and chondrocytes [50].

### 4.4. Mercury

Hg is situated in the 12th group and 6th period of the periodic table. The main sources of mercury contamination are coal combustion, gold mining and pesticides. It enters into the animal body through the consumption of contaminated feed and water [7]. The bioaccumulation of this element occurs mainly in the brain, kidney, liver, bones, and blood [25].

Mercury is cytotoxic, has the ability to induce cell apoptosis, disrupts the antioxidant system and increases the secretion of cytokines. In vitro studies have shown the toxic effect of organic and inorganic mercury on nerve cells [44]. Moreover, cell studies confirm that mercury negatively affects lymphocytes, myogenic cells and monocytes, leading to their apoptosis. Exposure to mercury causes increased levels of reactive oxygen species in cells which are correlated with cell death [44]. In turn, studies in mice have found that mercury affects the metabolism of fats in adipose tissue and may accelerate the development of obesity [46].

## 5. Monitoring of Environmental Contamination with Toxic Metals

Toxic metals are a common environmental pollutant, whose growth has increased significantly as a result of human activities. It is necessary to monitor their level in the environment and in animal tissues, mainly in the liver and kidneys, where they accumulate. Pollution monitoring aims to estimate human hazards, identify environmental contamination, and assess an area contaminated with metals [53]. Moreover, the determination of the level of metals in the tissues of animals enables the assessment of environmental pollution. For this purpose, biological methods, including bioindicators are used [53].

The exposure of animals to toxic metal poisoning is mainly influenced by feeding of the animals with feed that may be contaminated with substances containing toxins, such as pesticides and metals [54]. Therefore, it is necessary to monitor the contamination of animal feeds with toxic metals. Feed materials growing in polluted areas exposed to industrial emissions or contaminated soil show a high level of contamination. Control testing of the animals’ feed makes it possible to determine the levels of contamination and to control them [55].

Toxic metals are not biodegradable. They accumulate in the soil and enter the food chain with the soil. Apart from minerals, plants also take up toxic elements from the soil, which then enter into animal fodder, causing the intoxication of animal tissues, which may be a direct threat to humans, because these animal products are commercially available on the market as food [56].

Since 2006, there has been a lack of international standards for numerous toxic metals (e.g., Cu, Zn, As, Cr, Se, Mo) in food products like meat or milk, manufactured for humans, according to their potential risk of an adverse impact on human health [57]. Nevertheless, the observations involving the accumulation of toxic metals in tissues and organs have also been documented by Kołacz et al. (2017) [58], especially in the muscles, blood, liver and milk of cows, which pose a significant risk to the health of consumers. It was also found that literature data related to the influence of environmental pollutants (including metals considered as toxic) on metabolic changes in cattle (especially the metabolism of cows’ liver, e.g., the enzymatic activity of AST, LDH or GTP) are rare [58]. Furthermore, Kołacz et al. (2020) [59] confirmed that the contamination of plants, as well as animals, is an important aspect of animal husbandry affected by environmental pollutions, as well as by human nutrition policy, especially from Cu and Zn. Moreover, it was noted that the cooper easily binds to proteins and the excess of this metal may cause the effect of growth retardation, bone diseases, anaemia, teratogenesis or even Wilson’s disease or idiopathic copper toxicosis [59]. Kupczyński et al. (2017) [60] found that the high levels of microelements in the nutrition of ruminants may cause their bioaccumulation and toxic effects in the organism. It has also been reported that copper contributes significantly to the reduction of nutrient digestibility, lipid peroxidation, and causing oxidative stress in animals [60], which is greatly significant for animal welfare and for the quality of animal products [61]. It is also worth noting the fact that the copper deficiency in the organism may cause metabolic and immune disorders in humans and animals, which was confirmed by Harada et al. (2020) [62]. Zinc is also known as a crucial element of the human health status, being responsible for protein synthesis or cell growth, similar to growth and sex hormones [59]. It was also notes that the adequate Zn content is necessary for normal skeletal condition, reproductive functions and enzyme reactions. Nevertheless, an increased presence of zinc from the environmental sources is a serious risk affecting animals and products of animal origin. As it was mentioned by Kołacz et al. (2020) [59] that zinc present in an animal body interacts in metabolic processes with other metals (Cu, Fe and Ca), affecting blood circulation and also resulting in mental disorders which may be an effect of metabolic disturbances and a major cause of toxic effects in humans. The heavy metals (especially Cd and Pb) do not participate directly in redox reactions, and the effects of their toxicity reduce the effectiveness of cellular antioxidants [58]. It was also noted that both Cd and Pb may have a significant impact on mitochondria damage, or generate active forms of oxygen [58].

Despite numerous studies related to the intoxication of heavy metals in farm animals, taking into account their tissues reaching the food market, the connections related to the exposure of animals to toxic environmental factors and the identified poisoning or deficits of most of these elements in humans have not been directly proven, which leaves wide possibilities for future scientific research. For the determination of metals, anodic stripping voltammetry (ASV), atomic absorption spectroscopy (AAS) technology, optical emission spectrometry with plasma coupling (ICP-OES), or inductively coupled plasma mass spectrometry (ICP-MS) are used. These technologies show the greatest sensitivity and enable the detection of several metals at the same time [26]. Toxic metal biomonitoring can be performed by testing urine, blood, plasma, or hair. Urine collection may be used to determine the biologically effective dose biomarkers and the environmental intradose. Metals such as chromium, arsenic, and cadmium are excreted in the urine [63]. Urine samples for testing are collected for 24 hours to obtain results of their concentration in the body and to have an overview of excretion [26]. Moreover, specific gravity (U-SG) and urine creatinine (U-Cre) are used [63].

A urine test is a good way to identify As and Cr poisoning. Mercury is excreted in the feces and urine. In the case of lead, high concentrations are found in blood, hair, and urine [36]. Arsenic can also be detected in the blood up to a few hours after exposure, and in feces and keratin tissues where it accumulates [8,26]; arsenic is excreted from the body in feces and urine [8]. Blood is analyzed for lead, mercury, and cadmium. In turn, hair and fur are associated with the detection of arsenic and organic forms of mercury, which can be detected up to two weeks after exposure [26]. To diagnose cattle poisoning with toxic metals, milk can be tested, which is an indirect indicator of environmental pollution. These metals, mainly arsenic, cadmium, nickel, and lead, enter into animal organisms in feed and water, and then are excreted into cow’s milk [18].

Most of the toxic metals bioaccumulate in the internal organs, therefore it is possible to establish their levels in the body, postmortem [26]. The detection of contamination in animal feed contributes to the safety of human food. The concentrations of cadmium and mercury in animal feed are considered highly toxic at concentrations above 10 ppm, while for cobalt, copper, lead, molybdenum, or barium, concentrations above 40ppm are toxic [64]. Besides testing feed, animals’ blood, fluids and tissues should also be tested for toxic metals [64]. Various spectrometric techniques are used to identify metals in bovine tissues. These include atomic absorption spectrometry in a graphite furnace, optical emission spectrometry with inductively coupled plasma, and mass spectrometry. These techniques have low metal detection limits, as well as multimetal detection [65]. In North America, according to McGeehan et al. (2020) [64], these metals in the feed and samples are detected by mass spectrometry.

The Commission of the European Communities has defined the maximum residue levels (MRLs) of lead and cadmium (mg Cd/kg) as 0.1 and 0.05 in meat, 0.5 and 0.5 in liver, and 0.5 and 1.0 in bovine kidneys. In the case of Hg, the European Union has no legal restrictions on meat products. Moreover, the MRL of As in milk is defined as 0.1 (mg/kg). [65].

## 6. Preventing the Bioaccumulation of Toxic Metals

To restore the utility of soils, and thus indirectly reduce the exposure of animals and humans to toxic metals, soil remediation is used [52]. Soil remediation techniques are based on biological or chemical techniques [38]. Bioremediation is used to purify soil and water from toxic metals that pollute the environment. This involves the biological restoration of utility values of contaminated areas with the use of microorganisms and plants. By using *Bacillus* spp. or *Pseudomonas aeruginosa*, soil can be reclaimed from copper content. In turn, phytoremediation is used to remove cobalt from water, soil, and sediment. This immobilizes the toxic metals on plants thus preventing the spread of said metals. Bacteria symbiotic with these plants have the ability to adsorb these pollutants. Another method is rhizofiltration, which removes metals from water via plant roots and then accumulates them [38]. Chemical techniques include the reduction of the bioavailability of metals [38].

Environmental contamination with toxic metals results in the contamination of animal feed [54]. After the consumption of such feed, metals enter into the body of the animals and are often excreted in the animal’s feces and urine [66]. Animal manure is used as a fertilizer due to its rich composition. It contains proteins, polysaccharides, or minerals. However, it may also contain toxic metal contaminants [67]. To reduce pollution, composting is used, which reduces the risk of environmental contamination by the passivation of metals [66]. It consists of reducing the mobility and bioavailability of metals [5]. This is achieved by changing the physicochemical properties, such as fulvic acid (FA) and pH, as well as the use of microorganisms during composting. FA forms complexes with heavy metals of low molecular weight, and the alkaline reaction precipitates toxic metals and thus reduces their mobility in the environment. Moreover, the use of microorganisms results in the adsorption and oxidation of these metals [5,66].

According to Li et al. (2020) [67], composting manure for recycling generates significant amounts of greenhouse gases. Manure can be used as biomass for energy production as it has an energy value similar to that of wood waste. During energy production, which involves the thermal treatment of biomass, toxic metals are stabilized, which reduces their toxic effects. According to Li et al. (2020) [67], the incineration of manure transforms toxic metals with a high toxicity into a stable fraction with low bioavailability. During combustion, most of the metals remain in the solid phase of low toxicity. Arsenic, mercury, and lead are not in the solid fraction. This process prevents metal contamination, and is also a method for producing energy [67].

Landfills also contribute to environmental pollution with toxic metals. As a result of the degradation of waste or the access of water to it, leachate is generated, which can contaminate the soil and groundwater [68,69]. The metal-containing leachate is then a source of contamination. Landfill soils are reclaimed using phytoremediation. This method is based on the use of plants that accumulate metals by removing them from soil or water. The success of phytoremediation is influenced by the ability of plants to absorb and accumulate metals, as well as their availability to the plants. Moreover, biochar shows the ability to improve soil quality and significantly reduce the bioavailability of toxic metals [68]. Biochar is produced by the pyrolysis of biomass [69].

Sewage sludge is most often deposited in landfills; however, it is associated with a potential to contaminate groundwater and soil. Then, the optimal method for the disposal of sewage sludge is pyrolysis, and the resulting biochar can be used to improve the properties of the soil and its fertility [69]. The addition of biochar to sewage sludge effectively reduces the risk of metals that are not removed during sewage treatment [67]. The pyrolysis of sewage sludge to biochar and the further use of biochar reduces pollution in sewage sludge [67,69]. According to Penido et al. (2019) [69], this reduces the bioavailability of cadmium and leads to plant development. Furthermore, biochar increases the pH of the soil and reduces the bioavailability of toxic metals [69].

## 7. Conclusions

Heavy metal pollution is common in the environment and can come from natural sources such as forest fires and volcanic eruptions, as well as from anthropogenic sources: mines, smelters, or refineries [7]. These elements are toxic to living organisms and internal organs and can accumulate in living organisms [40]. They can negatively affect both female and male fertility [40]. The chronic exposure of cattle to toxic metals can cause embryotoxicity, disturbances in spermatogenesis, and oocyte development [7]. It is important to monitor environmental pollution caused by toxic metals [6].

There is a need for more research into the effects of toxic metals on the fertility of cows and bulls.

## Figures and Tables

**Table 1:** Toxic metals and their influence on the reproductive functions of cattle.

Toxic Metal	Source	Penetration into Body	Bioaccumulation	Toxic Effect	References
Pb	Mining Pb-acid batteries, gasoline in petrol, pigments in paints, electronic wastes, pesticides, fires, volcanic eruptions	Ingestion of contaminate feed and water	Liver, kidney, brain, bones, testes, epididymis, seminal vesicle, ejaculate, follicle fluid	Male: Damage to sperm reduces spermatozoa count and motility, azoospermia, asthenozoospermia, morphological abnormalities of sperm and disorders in prostatic function Female: Abortion, infertility, pregnancy, hypertension, premature calving, follicular artesia	[7,28,35,36]
Hg	Coal combustion, gold mining, pesticides, volcanic eruptions, wildfire	Ingestion of contaminated feed and water, inhalation	Brain, kidney, boses, blood, hair, liver	Damage of testicular cells reduces semen quality and spermatogenesis, increases oxidative stress, and damages sperm membrane Fetotoxic, Ataxia, neuromuscular incoordination, convulsions	[6,25,30,36]
As	Mining, smelting, combustion of fossil fuel, production of chemotherapeutic drugs of cancer, production of glass, volcano eruptions	Ingestion of contaminated feed and water	Liver, heart, lungs, kidney, blood, keratin tissues	Decrease of testosterone, LH, FSH, increase of cortisol, Leydig cells, atrophy Ataxia, anorexia, diarrhea, hepatoxic	[6,8,26,36]
Cd	Combustion of fossil fuels, Ni-Cd batteries, mining and smelting operations, volcano eruptions, forest fires, dust storm, erosion	Ingestion of contaminate feed and water	Kidney, lungs, bones, testes, epididymis, seminal vesicle, ejaculate	Male: Abnormalities of sperm caused separated tail of sperm, a decrease in the mass of the testes, changes in Sertoli cells and seminal tubules, damage sperm DNA, decreases antioxidant status in semen, Female: reduces ovarian function, suppresses oocyte maturation, cytotoxic to oocytes, reduces oocytogenesis	[6,19,35,37]
Co	Mining, incinerators, leaching, hard metal production, batteries	Through the digestive system	Bones	Reduces oocyte maturation, ovulation, gametogenesis,	[37,38]
Cu	Mining, cement production, coatings and painting, transport, metal mining	Ingestion with contaminated feed	Lung, spleen, liver, kidney, intestine	Reduces mitochondrial activity, induces apoptosis of cumulus cells, damages ovaries, causes abnormalities of sperm, causes the separated tail of sperm, reduces sperm motility	[6,36,39]
Mn	Transport, soil fertilizers, waste management, industry	Through the digestive system or respiratory system	Follicular fluid,	Damage acrosome and plasma membranes reduce fertility	[6,39]
Ni	Coal combustion	Through the digestive system	Kidneys	Causes separated flagellum of spermatozoa and reduces sperm concentration and motility	[6,40]
Cs	Radiography, gamma radiation	Through ionizing radiation	Testicles, vas deferens	Reduces fertility causes abnormal sperm and azoospermia; damages of spermatogonia impairs male and female germinal cells, induces testicular cancer, the radioactivity of cesium is transferred from cow to the fetus via the placenta	[6,41]
Cr	Leather tanneries, textile industry, steel industry, coal combustion, wood burning, production of dyes and wood preservatives	Ingestion with contaminated feed	Liver, kidney,	tumors in stomach	[40,42]

## Data Availability

Not applicable.
